# Investigating the Interaction of Fe Nanoparticles with Lysozyme by Biophysical and Molecular Docking Studies

**DOI:** 10.1371/journal.pone.0164878

**Published:** 2016-10-24

**Authors:** Zahra Aghili, Saba Taheri, Hojjat Alizadeh Zeinabad, Leila Pishkar, Ali Akbar Saboury, Arash Rahimi, Mojtaba Falahati

**Affiliations:** 1 Department of Biology, Islamic Azad University, Science and Research Branch, Tehran, Iran; 2 Department of Biology, Islamshahr Branch, Islamic Azad University, Islamshahr, Iran; 3 MEMS & NEMS Lab, Department of Electrical and Computer Engineering, University of Tehran, Tehran, Iran; 4 Young Researchers and Elite Club, Islamshahr Branch, Islamic Azad University, Islamshahr, Iran; 5 Institute of Biochemistry and Biophysics (IBB), University of Tehran, Tehran, Iran; 6 Center of Excellence in Biothermodynamics, University of Tehran, Tehran, Iran; 7 Department of Nanotechnology, Faculty of Advance Science and Technology, Islamic Azad University of Pharmaceutical Sciences (IAUPS), Tehran, Iran; Kermanshah University of Medical Sciences, ISLAMIC REPUBLIC OF IRAN

## Abstract

Herein, the interaction of hen egg white lysozyme (HEWL) with iron nanoparticle (Fe NP) was investigated by spectroscopic and docking studies. The zeta potential analysis revealed that addition of Fe NP (6.45±1.03 mV) to HEWL (8.57±0.54 mV) can cause to greater charge distribution of nanoparticle-protein system (17.33±1.84 mV). In addition, dynamic light scattering (DLS) study revealed that addition of Fe NP (92.95±6.11 nm) to HEWL (2.68±0.37 nm) increases suspension potential of protein/nanoparticle system (51.17±3.19 nm). Fluorescence quenching studies reveled that both static and dynamic quenching mechanism occur and hydrogen bond and van der Waals interaction give rise to protein-NP system. Synchronous fluorescence spectroscopy of HEWL in the presence of Fe NP showed that the emission maximum wavelength of tryptophan (*Trp*) residues undergoes a red-shift. ANS fluorescence data indicated a dramatic exposure of hydrophobic residues to the solvent. The considerable reduction in melting temperature (*T(m)*) of HEWL after addition of Fe NP determines an unfavorable interaction system. Furthermore circular dichoroism (CD) experiments demonstrated that, the secondary structure of HEWL has not changed with increasing Fe NP concentrations; however, some conformational changes occur in tertiary structure of HEWL. Moreover, protein–ligand docking study confirmed that the Fe NP forms hydrogen bond contacts with HEWL.

## Introduction

Unique metallic nanoparticles have shown great prospect in the field of nanobiotechnology and nanobiomedicine for the past few decades due to a variety of intriguing characteristics such as size, biocompatibility, high surface to volume ratio, functionalization, biomimetic features, and biodegradability [1, 2]. Among the metallic nanoparticles, iron nanoparticles (Fe NPs) have attracted a great potential in a wide variety of biotechnology and biomedical applications such as cellular targeting, labeling, imaging and drug delivery owing to the fact that they exhibit novel magnetic features and dramatic biocompatibility characteristics [3–5]. However, little is known about potential side effects of Fe NP on cellular and subcellular systems, which is an obstructive point to the development of Fe NPs on nanomedicine and nanobiotechnology. In particular, the interaction of Fe NPs with cells, subcellular organelles, and biological macromolecules such as DNA, lipid, and proteins is still an enigma [6–8]. Toxicity and biodistribution of these nanoparticles have been rarely understood so far [8, 9]. Fe NPs could be used as a potential candidate in MRI, drug delivery, magnetic separation and immobilization of biosubstances [4, 5]. Hence, the demand of biomedicinal application of Fe NP is gradually increasing. To understand the mode of action between NPs and bio-macromolecules a mechanism of interaction and following structural changes of protein should be considered during the study of interaction of Fe NPs with the proteins.

Our report presented a detailed enquiry on the mechanism of interaction between Fe NPs and a model biological protein (lysozyme). Hen egg white lysozyme (HEWL) is a relatively small enzyme with 129 residues that catalyzes the hydrolysis of specific kinds of polysaccharides in outer cell walls of bacteria, damages their integrity and the bacteria's viability. Based on the CATH structure classification database, HEWL has been classified as “mostly α*-*helix” [10]

In the present research, the interaction of Fe NP and HEWL was mainly investigated with zeta potential and DLS measurements, fluorescence spectroscopy, CD spectroscopy, and docking study. Zeta potential and DLS studies was employed to determine the potential suspension of nanoparticle-protein system. The fluorescence quenching constants, association constants, number of binding sites, thermodynamic parameters, and microenvironmental changes of aromatic residues of the HEWL upon interaction with Fe NP HEWL were determined using fluorescence spectroscopy. By determining these data, we can reveal the quenching mechanism and primary dominant interactions between the Fe NP and HEWL. In addition, the mechanism of the reaction was further investigated by CD spectra which can demonstrate secondary and tertiary structural changes of HEWL upon interaction with Fe NP. Computational docking study was done to investigate the dominant interactions between protein and Fe NP. The findings of this paper will not only play a pivotal role on the toxicity of Fe NP and the binding mode of Fe NP and proteins but also, bring complementary information for the application of Fe NP in biotechnological, medicinal, and pharmaceutical fields.

## Materials and Methods

### Materials

HEWL from with catalog number of L7651 was purchased from Sigma-Aldrich (USA). Iron nanoparticle nanopowder, 35–45 nm particle size, 99.5% trace metals basis with a CAS number of 7439-89-6 was purchased from Sigma-Aldrich Company (St. Louis, USA).

### Methods

#### Suspension of iron nanoparticle

Fe NP was suspended in deionized water pH 7.8 (adjusted by NaOH and HCl), and was analyzed by DLS and zeta potential measurements.

Zeta potential and hydrodynamic radius values of HEWL, Fe NP and HEWL/ Fe NP (mass ratio of 1:1) were done with DLS (Brookhaven Instrument, Holtsville, NY 11742–1896, USA) at room temperature. Three run was carried out to quantitatively express the average zeta potential of each solutions.

#### Fluorescence quenching measurement

The intrinsic fluorescence spectroscopy of HEWL in the presence of varying concentrations of Fe NP at different temperatures (25, 37, and 42°C) was carried out on Spectrofluorimeter, MPF-4 model (Hitachi, Japan) by a rectangular quartz cell with 1 cm path length. The excitation wavelengths were set at 285 nm. Slit widths of both excitation and emission were set to 10 nm.

The HEWL concentration in phosphate buffer (pH 7.8, 50 mM) was fixed at 4 μM, and the fluorescence intensity changes of HEWL were recorded in the presence of varying concentrations of Fe NP ranging from 2, 4, 8, 15, 30, 50 and 100 μM. All experiments were carried out in triplicates and the fluorescence intensity values of Fe NP and buffer sample at applied concentration and wavelengths were almost zero.

#### Synchronous fluorescence spectroscopy

The synchronous fluorescence spectra of HEWL (4 μM) were monitored in the presence of varying concentrations of Fe NP (2, 4, 8, 10, 15, 30, 50 and 100 μM). Indeed, simultaneous scanning of the excitation and emission monochromators was carried out, while a constant wavelength interval of Δλ, 15 nm and 60 nm was kept to investigate the microenvironmental changes of tyrosine (*Tyr*) and tryptophan (*Trp*) residues, respectively. Slit widths of both excitation and emission were fixed at 10 nm.

#### ANS-fluorescence study

The extrinsic fluorescence intensity of the HEWL (4 μM) upon interaction with varying concentrations of Fe NP (2, 4, 8, 10, 15, 30, 50 and 100 μM) was evaluated using 8-anilino-1-naphthalenesulfonic acid (ANS) (100 μM) as a fluorescent probe at room temperature. The excitation wavelength was fixed at 350 nm and the fluorescence emission was read at 400 to 600 nm in a Spectrofluorimeter, MPF-4 model (Hitachi, Japan). Slit widths for both excitation and emission were fixed at 10 nm. The samples were incubated in dark at room temperature for 15 min and stirred gently to avoid aggregation.

All experiments were done in triplicates and the ANS emission intensities of Fe NP and buffer sample at above concentrations and wavelengths were negligible.

#### *T(m)* measurement

Fluorescence intensity changes of HEWL and HEWL/ Fe NP complex were monitored against heat denaturation at excitation wavelength of 285 nm using a Hitachi Spectrofluorimeter, MPF-4 model (Hitachi, Japan) equipped with a thermostatically controlled cuvette holder at scan rate of 1°C/min. HEWL and Fe NP/cyt c complex at a concentration of 4μM and 4/50 μM, respectively were dissolved in phosphate buffer (pH 7.8, 50 mM). The heating curves were subtracted from Fe NP and buffer solutions baselines.

#### Far and near circular dichroism spectroscopy

The far (190 to 260 nm) and near (260 to 340 nm) CD spectra of HEWL in the presence of varying concentrations of Fe NP were monitored. The CD measurements were done on an Aviv model 215 Spectropolarimeter (Lakewood, NJ, USA) at room temperature using a rectangular quartz cell with 0.2 and 1 cm path length for far and near CD, respectively. All measurements were run in triplicates and reported as the average and also were subtracted from buffer sample. The secondary structural changes of HEWL with a concentration of 15 μM (pH 7.8, 50 mM phosphate buffer) were recorded with addition of varying concentrations of Fe NP ranging from 5–150 μM. The α-helix content of HEWL in the presence of Fe NP was calculated using CDNN software from mean residue ellipticity (MRE) values at 208 nm by the following Eq (1):

$$\alpha -\mathrm{Helix}\left(\%\right)=\frac{\left(-\mathrm{MRE}208–4000\right)}{\left(33,000–4000\right)}\times 100$$ equation (1)

Whereas MRE_208_ is the measured MRE value at 208 nm, 4000 is the MRE of the β-form and random coil structure at 208 nm, and 33,000 is the MRE value of the pure α-helix at 208 nm.

MRE_208_ can be calculated from the following Eq (2):

$$\mathrm{MRE}\;208=\frac{ϴ\left(\mathrm{m deg}\right)}{\mathrm{Cp nl}10}$$ equation (2)

Where, Ɵ is observed CD, C_p_ is the molar concentration of the protein, n is the number of amino acid residues and l is the path length of the quartz cell.

Near CD spectra of HEWL (75μM) (at wavelength of 260–340 nm) were also recorded in the presence of varying concentrations of Fe NP (25–75 μM).

The ellipticity values of Fe NP and buffer sample at applied concentrations were negligible in far and near CD experiments and also were subtracted from HEWL samples.

#### Molecular docking

In order to evaluate the water-covered nanoparticle affinity to bind the protein, at first the geometries of isolated Fe with 13 atoms in a cluster was optimized as a model of iron nanoparticle using the CASTEP module [11] in Materials Studio 5.0 developed by Accelrys Software Inc. As the Fe NP in the optimized situation in the formed cluster has 12 surface atoms, we considered one adsorbing water molecule for every surface atom. Steric effect between water molecules causes lowering binding energy for complexes with further H_2_O molecules.

Electron-ion interactions were modeled using local density approximation (LDA) functional and ultra soft pseudo potentials [12]. Then 12 H_2_O molecules were added to the cluster and geometry procedure was repeated at the same level of theory. A molecular docking was performed using HEX 6.3 software [13].

## Results and Discussion

### Zeta potential and size measurements

Zeta potential is a scale of stability of suspended particles. A higher electric charge on the surface of particles will reduce their tendency to aggregation due to the potent repellent forces among them. It is well documented when zeta potential values are above 30 mV, in the range of 5 mV to 30 mV, and below 5 mV, they indicate a good stability, short term stability, and fast aggregation of particles, respectively.

The effect of Fe NP addition on zeta potential and size distribution of HEWL/ Fe NP system was studied through a zeta potential and dynamic light scattering measurements. The results showed that the addition of Fe NP (zeta potential of 6.45±1.03mV) to HEWL (zeta potential of 8.57±0.54 mV) could increase the zeta potential of Fe NP /HEWL systems with a value of 17.33±1.84 mV (Table 1). Also to confirm the zeta potential outcomes, DLS measurement were done and the hydrodynamic radii of HEWL, Fe NP and HEWL/ Fe NP solutions were measured as 2.68±0.37, 92.95±6.11and 51.17±3.19 nm, respectively (Table 1).

**Table 1:** Zeta potential and hydrodynamic radius of HEWL, Fe NP and HEWL/ Fe NP systems.

System	Zeta potential (mV)	Hydrodynamic radius (nm)
**HEWL**	8.57±0.54	2.68±0.37
**Fe NP**	6.45±1.03	92.95±6.11
**HEWL- Fe NP**	17.33±1.84	51.17±3.19

**Fig 1:**
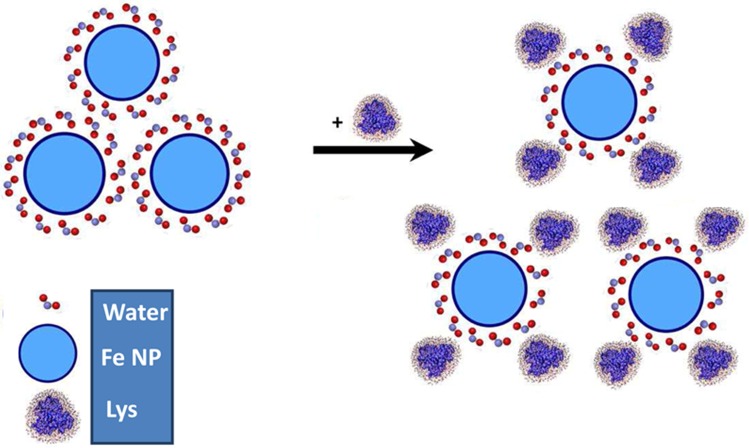
Suspension potential of protein/nanoparticle system. Schematic representation of enhancing suspension potential of protein/nanoparticle system via absorption of proteins on the Fe NP surfaces.

Te larger size of Fe NP in solution relative to the nominal size of company may be attributed to the tendency of Fe NP to aggregation and presence of water molecules on the NP surface. While, in general, the loss of structure of protein occurs upon binding to NPs and is considered as a disadvantage of probable use of nanoparticle, there is a potential advantageous consequence too. Promising application of proteins and NPs include enhancing suspension potential of protein/NP system via elevation of complex charges of surface and preventing aggregation (Fig 1).

The size of nanoparticle also can play a pivotal role in the interaction mechanism and charge distribution of the protein/nanoparticle system. As larger nanoparticles exhibit a higher average aggregation tendency, therefore the effect of protein on suspension potential of nanoparticles could be more pronounced.

Proteins can attach on the nanoparticle surfaces and hinder nanoparticle-nanoparticle interaction and subsequent aggregation. One of the most disadvantageous features of nanoparticles in the biological systems is refereed to their tendency to aggregation. Based on these data it can be suggested that interaction of nanoparticles with proteins can enhance the potential solubility of nanoparticles and actuate the beneficial usage of nanoparticles in biological application. Therefore the optimum concentration of nanoparticles and protein should be precisely considered for further designing and developing of nanoparticles-based drug carriers.

### Fluorescence spectroscopy experiments

**Fig 2:**
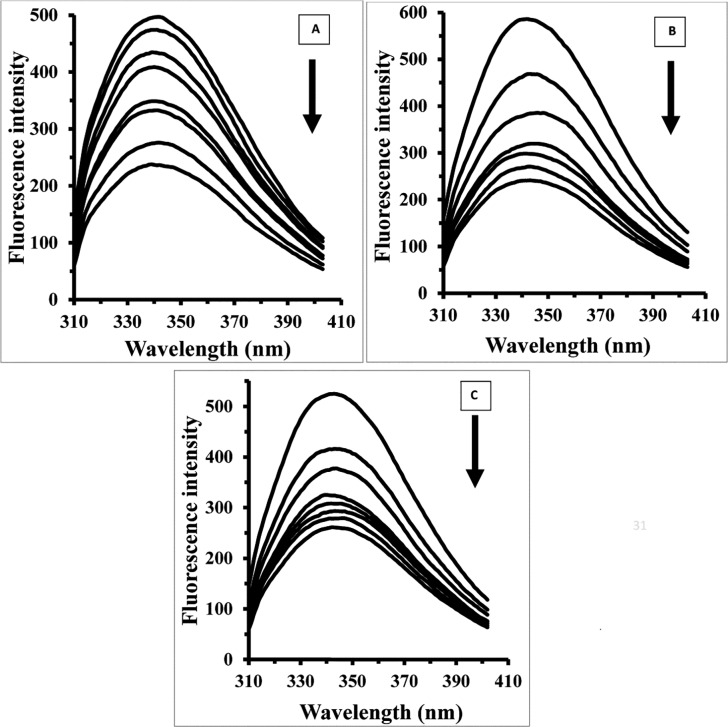
Fluorescence studies of HEWL upon addition of Fe NP. The HEWL concentration in phosphate buffer (pH 7.8, 50 mM) was 4 μM, and the fluorescence intensity changes of HEWL were recorded in the presence of varying concentrations of Fe NP (2, 4, 8, 15, 30, 50 and 100 μM) at three temperatures of 25 (A), 37 (B) and 42°C (C).

The fluorescence spectroscopy experiments were designed to consider structural changes of HEWL in the presence of varying concentrations of Fe NP. Fig 2 shows that HEWL provides strong fluorescence intensity at 340 nm when exciting at 285 nm. With gradual titration of Fe NP the fluorescence intensity of HEWL is shown to be quenched. To determine the nature of quenching mechanism of the fluorescence emission of HEWL upon interaction with Fe NP, the Stern–Volmer (SV) equation has been applied as following [14–16]:

$$\frac{\mathrm{Fo}}{F}=1+\mathrm{Kq\tau}0\left[Q\right]=1+\mathrm{KSV}\left[Q\right]$$ equation (3)

**Fig 3:**
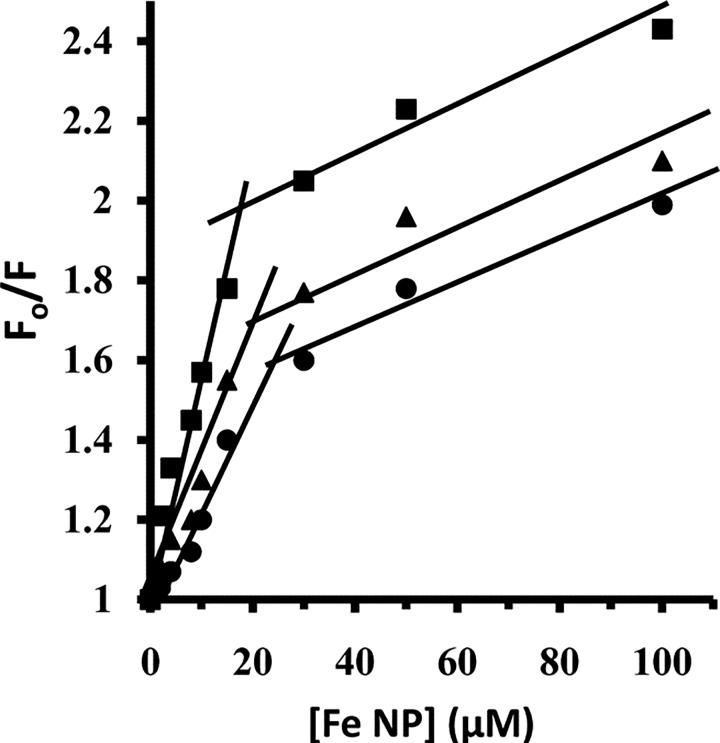
Stern–Volmer plot. Stern–Volmer plot for the binding of HEWL with Fe NP at 25 (●), 37 (▲) and 42°C (■).

Where F_o_ and F represent the steady state fluorescence intensities in the absence and presence of quencher, respectively. K_SV_ donates the Stern–Volmer quenching constant, [Q] is the concentration of quencher, kq is the bimolecular quenching rate constant, and τ_0_ is the biomolecular fluorescence lifetime in the absence of quencher, which is considered to be 6 ns [17–19]. As shown in Fig 2, the SV plot is not linear in nature. The deviation from linearity of SV plot indicates that both dynamic and static type of quenching occurs in the interaction of HEWL with Fe NP [20]. From linear plots of F_0_/F against [Q], two independent K_SV_ values are calculated from 0 to 15 μM and from 15 to 100 μM of Fe NP (Fig 3). As K_SV_ = k_q_τ_0_, where τ_0_ = 6 ns, the kq_1_ is estimated and found to be 48.7 ×10^l1^ M^−1^ s^−1^ at 298 K till 15 μM of Fe NP (Table 2). The calculated kq_1_ is much greater than the maximum collision quenching constant (10^10^ M^−1^ s^−1^) [21, 22]. After elevation of Fe NP concentration over 15 μM, the kq_2_ is calculated about 58.2 ×10^l0^ M^−1^ s^−1^ (Table 2). This indicates at high concentrations of Fe NP the kq_2_ is an order of 10^10^ M^−1^ s^−1^ and therefore dynamic mode is mainly involved in the mechanism of quenching process. So it can be concluded that HEWL quenching by Fe NP is primarily of static type initiated by ground state complex formation and followed by a dynamic collision. A curve model of linear Stern-Volme plot also indicates all fluorophores are not equally accessible to the quencher, otherwise a linear Stern-Volmer plot is found [23, 24].

**Table 2:** Stern-Volmer binding parameters of interaction between HEWL and Fe NP.

T (K)	K_SV1_ (M^−1^)	K_SV2_ (M^−1^)	kq_1_(M^−1^s^-1^)	kq_2_(M^−1^s^-1^)
**298**	29.25±2.43 ×10^3^	34.92±4.27×10^2^	48±3.78.76×10^11^	58.2±4.93×10^10^
**310**	65.54±5.85×10^3^	84.30±7.96 ×10^2^	10.92±1.27×10^12^	14.05±1.13×10^11^
**315**	75.10±8.37×10^3^	69.98±5.88×10^2^	12.51±1.72×10^12^	11.66±1.05×10^11^

This obtained data may reveal that at low concentration of Fe NP, HEWL is still in the form of globular structure and polar residues on the surface of protein could establish new and additional linkages with water molecule on the surface of Fe NP. However, when concentration of Fe NP is increased, it may induce more conformational changes of protein and provides an unfavorable environment for protein-NP interaction. In other words, protein has experienced some conformational changes and following residual reorientation at higher concentration of Fe NP which will be efficient in preventing polar-polar interaction between surfaces of protein and NP. To further investigate these mechanisms, binding modes during interaction of Fe NP with HEWL was considered.

### Determination of the preferential binding modes

Upon interaction of nanoparticles with the protein surface, the binding constant (K_b_) and the number of binding sites per protein molecule (n) for protein-nanoparticle system can be determined based on the following equation [25, 26]:

$$\log\frac{\mathrm{Fo}-F}{F}=\mathrm{log Kb}+\mathrm{nlog}\left[Q\right]$$ equation (4)

The values of K_b_ and n for HEWL upon interaction with varying concentrations of Fe NP were determined from the intercept and slope of log [F_o_-F/F] versus log[Q] plots at different temperatures and were summarized in Table 3. As seen from Table 3, the impact of increasing the temperature of a system is to reduce the K_b_ values of HEWL/ Fe NP system. This revealed that after increasing of temperature, tertiary structural changes of protein can occur and Fe NP has higher affinity for HEWL at 298 K than 310 K. Therefore, the K_b_ value of HEWL/ Fe NP system reduces with elevation of temperature. When the temperature is raised, it causes a negative effect on the interaction of Fe NP with HEWL. Moreover, as shown in Table 3, upon interaction of Fe NP with HEWL the number of binding sites per protein is ~ 0.46. Temperature- induced tertiary structural changes of HEWL has led to more unfolded conformation of HEWL backbone and subsequently displacement of accessible binding residues. This can be well documented by a decrease in number of binding site per protein for Fe NP at higher temperatures.

**Table 3:** Thermodynamic and binding parameters of interaction between HEWL and Fe NP.

System	T	K_b_	n	ΔG°	ΔH°	ΔS°	Reference
	(K)	(M^−1^)		(kJ/mol)	(kJ/mol)	(J/mol K)	
**HEWL- Fe NP**	298	105.07±7.45	0.56	-6.82±0.54	-90.89±7.16	-282.11±17.24	
	310	45.42±3.68	0.44	-3.43±0.45			
	315	07.52±0.51	0.21	-2.02±0.17			
**γ-Fe**_**2**_**O**_**3**_**-fibrinogen**	298	2.24 × 10^7^	1.06	−41.86	−72.33	−102.26	(30)
**NH**_**2**_**-Fe**_**3**_**O**_**4**_**-BSA**	298	69.5× 10^8^	1.5	-0.78	-1.99	-313.95	(31)
**Fe**_**3**_**O**_**4**_**-BSA**	298	1.05× 10^8^	1.3	-0.64	-1.28	-166.53	(31)
**Fe**_**2**_**O**_**3**_**-Hb**	298	6.1 × 10^5^	1.3	-32.9	-115.34	-281.56	(32)

### Thermodynamic parameters

The value and sign of thermodynamic parameters (Gibbs free energy change, *ΔG*^*o*^, enthalpy change, *ΔH*^*o*^, and entropy change, *ΔS*^*o*^) provide important information regarding the binding forces between proteins and nanoparticles.

Therefore, *ΔS*^*o*^, *ΔH*^*o*^, and *ΔG*^*o*^ were calculated to reveal the interaction forces in HEWL/ Fe NP system. The interaction forces between biological macromolecules and ligands may involve hydrogen bonds, van der Waals forces, ionic and hydrophobic interactions. According to thermodynamic parameters, if *ΔH*^*o*^ and *ΔS*^*o*^ values are negative, then hydrogen bonds and van der Waals interactions are the dominant forces between protein and nanoparticles. When *ΔH*^*o*^ and *ΔS*^*o*^ values are positive, hydrophobic interactions play important roles in the binding forces.

**Fig 4:**
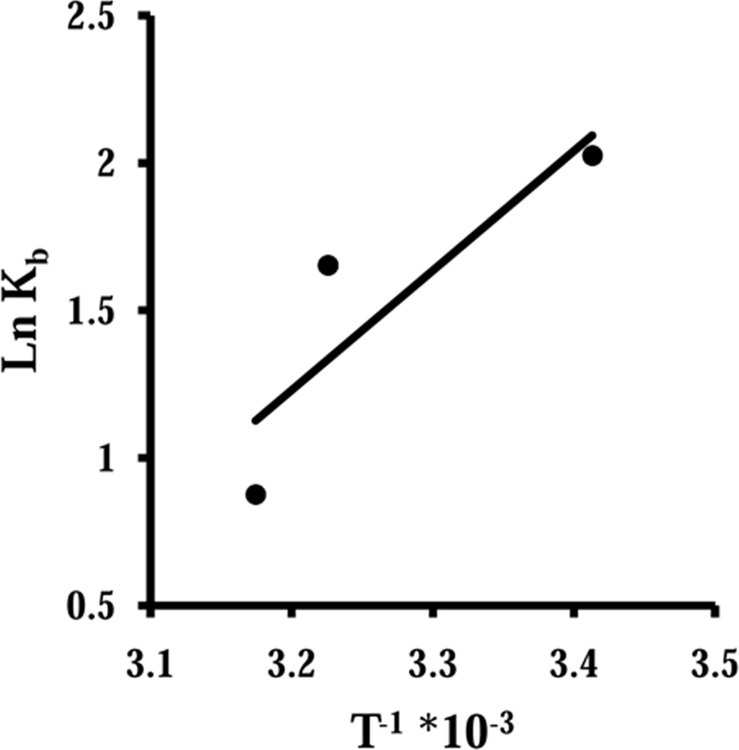
The plot of ln K_b_ versus 1/T. The plot of ln K_b_ versus 1/T yields *ΔH°* and *ΔS°* values.

When *ΔH*^*o*^ is negative and *ΔS*^*o*^ is positive, then electrostatic interactions are responsible for new and additional linkages between protein and nanoparticle [27, 28]. If *ΔH*^*o*^ and *ΔS*^*o*^ values remain almost constant over the temperature range examined, then *ΔH*^*o*^ and *ΔS*^*o*^ values can be determined from van't Hoff equation [29]:

$$\mathrm{ln Kb}=\frac{-\mathrm{\Delta H}{}^{\circ}}{\mathrm{RT}}+\frac{\mathrm{\Delta S}{}^{\circ}}{R}$$ equation (5)

where K_b_ is the binding constant at given temperature and R is the universal gas constant. According to Eq (5), the plot of ln K_b_ versus 1/T gives a straight line with a slope of *ΔH°* and Y-intercept of *ΔS*°. Hence, to determine *ΔH°* and *ΔS°* values, ln K_b_ versus 1/T were plotted for HEWL/Fe NP system (Fig 4) and these values are summarized in Table 3. Then, *ΔG°* values were determined from the Gibbs-Helmholtz equation:

ΔG°=ΔH°−TΔS° equation (6)

The calculated *ΔG°* values are also given in Table 3. In this study, the sign of *ΔG°*, *ΔH°* and *ΔS°* were all negative for the binding processes of Fe NP to HEWL. These negative values of *ΔH°* and *ΔS°* indicate that the potent binding forces in the binding of Fe NP with HEWL are hydrogen bonds and van der Waals interactions. In addition, from negative *ΔG°* values it can be concluded that the binding process of Fe NP with HEWL is spontaneous. Also with comparison of interaction between iron and iron oxide nanoparticle and different proteins, it was revealed that all species of iron nanoparticles bind to different kinds of proteins by means of hydrogen bonds [30–32]. In general, it seems that the presence of oxygen groups on the nanoparticle surfaces does not change the basic nature of Fe NP for interaction with proteins. Indeed, the nature of Fe plays a considerable role in the interaction mode and presence or absence of oxygen is not a remarkable factor in the interaction mechanism. This may be attributed to the presence of water molecules on the nanoparticle surface which can alter the properties of iron or iron oxide nanoparticles. It can be concluded that the water molecules on the surface of iron or iron oxide nanoparticles specifies the mode of interaction and the presence of oxygen and the kind of nanoparticles assign the degree of protein conformational changes.

### Synchronous fluorescence spectroscopy study

**Fig 5:**
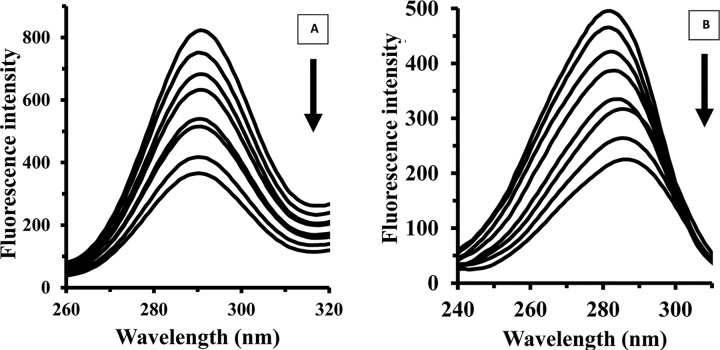
Synchronous fluorescence spectroscopy of HEWL upon addition of Fe NP. The synchronous fluorescence spectra of HEWL (4 μM) were recorded at interval of Δλ, 15 nm (A) and 60 nm (B) in the presence of varying concentrations of Fe NP (2, 4, 8, 10, 15, 30, 50 and 100 μM).

Synchronous fluorescence spectroscopy provides descent information regarding the micoenvironmental changes of *Trp* and *Tyr* residues. It simultaneously scan both excitation and emission monochromators while keeping a constant wavelength interval. This technique provides pivotal data to investigate the environment of chromophore (*Trp*, *Tyr*) by calculating the possible shift in the position of maximum emission wavelength, which is known as, λ_max_. These shifts are according with the alterations of the polarity around the chromophore residues [30, 32]. When the difference between excitation and emission wavelengths is fixed to 15 or 60 nm, the synchronous fluorescence can present the typical information of the micoenviromental changes of *Tyr* or *Trp* residues, respectively [33,34]. Therefore the synchronous fluorescence spectroscopy of HEWL in the presence of varying concentrations of Fe NP is demonstrated in Fig 5. As shown in Fig 5, the fluorescence of *Tyr* and *Trp* residues was almost strong and the position of λ_max_ of Tyr residues did not show any shift when Δλ was set to 15 nm. However, the λ_max_ of Trp residues were shifted profoundly toward longer wavelengths, i.e., a remarkable red shift of approximately 5 nm, when Δλ was set to 60 nm. These data present valuable information regarding the microenvironmental changes around the Trp residues. It seems Trp residues ship to a more hydrophilic residue and expose to the solvent when HEWL is affected by Fe NP.

### ANS-fluorescent experiment

**Fig 6:**
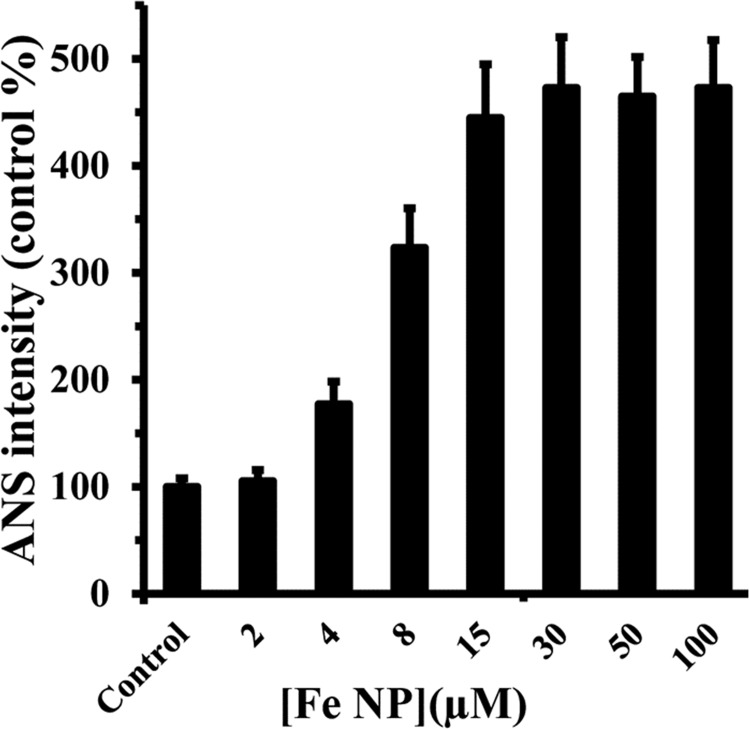
ANS fluorescence intensity of HEWL upon addition of Fe NP. The ANS (100 μM) fluorescence intensity of the HEWL (4 μM) was measured upon interaction with varying concentrations of Fe NP (2, 4, 8, 10, 15, 30, 50 and 100 μM). ANS fluorescence intensity of Fe NP was almost zero and was also subtracted from all samples.

1-anilino-8-naphthalene sulfonate (ANS) tends to bind to hydrophobic moieties on the surface of proteins. After addition of various concentrations of Fe NP to HEWL, the fluorescence intensity of ANS was measured using excitation wavelength of 350 nm. ANS does not normally tend to attach to folded structure of HEWL (normalized to 100% as control) (Fig 6). However with the addition of Fe NP to HEWL, a dramatic increase of the ANS fluorescence intensity was observed compared to the native HEWL, presumably was inspired by disposition of hydrophobic pocket of HEWL and exposing to a solvent accessible area (Fig 6). This enhancement of ANS intensity is started to be almost constant above 15 μM which is in good agreement with the Stern-Volmer analysis of Fe NP-HEWL interaction, stating that Fe NP interaction with HEWL surface is stronger in lower concentrations of nanoparticles relative to higher concentrations. Indeed interaction of Fe NP with HEWL could occur by means of polar-polar forces and this interaction is stable when protein is in globular form with distribution of hydrophilic polar residues on the surface of protein.

It was seen that at room temperature ANS did not usually attach to the native protein. This enhancement in ANS intensity upon addition of varying concentrations of Fe NP is likely attributed to the distinctive exposure of hydrophobic residues of protein during interaction with nanoparticle. In order to more support the rearrangement of protein structure in the presence of Fe NP, thermal stability measurement of HEWL in the absence and presence of Fe NP was done.

### Thermal stability measurement

**Fig 7:**
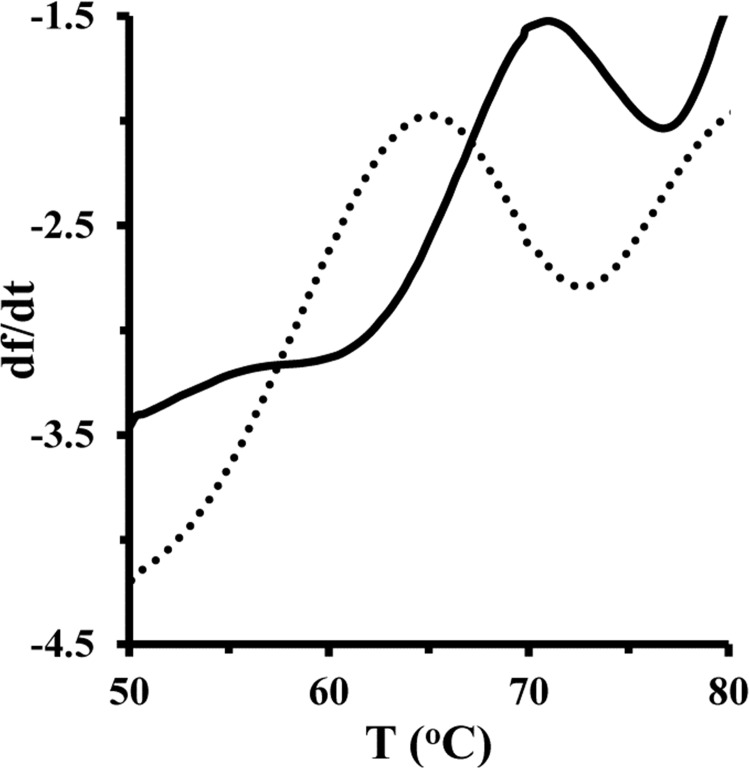
Melting temperature measurement of HEWL and HEWL/ Fe NP system. Melting temperatures of HEWL (4μM) (solid line) and HEWL/ Fe NP complex (4/50) (dashed line) were measured.

In order to evaluate the role of Fe NP in the thermal stability of HEWL, this experiment was carried out in the presence of a single concentration of Fe NP. Thermal stability of HEWL in the presence of Fe NP was studied by fluorescence spectroscopy and the outcome was expressed as the first derivatives of intensity. Fig 7 shows *T(m)* of the protein was about 71.25°C. However, addition of Fe NP in the protein solution results in the reduction of *T(m)* of HEWL about 65.48°C. This outcome indicated that the presence of Fe NP may result in the formation of less stable species of HEWL relative to the pure protein.

The interaction between nanoparticles and proteins normally causes alterations in protein thermal stability with changes in the melting temperature. These changes can be induced by modifications in protein conformation and structural flexibility, which is provided by nanoparticle interaction. Indeed, Fe NP ligand induces a decrease in *T(m)* of HEWL. This reduction of *T(m)* may be attributed to the conformational changes of protein structure toward a more unpacked structure. In other words, Fe NP establishes new and weak linkages with protein surface which rupture the folded structure of protein and corresponding bonds inside the protein.

### Analysis of secondary and tertiary structural change

**Fig 8:**
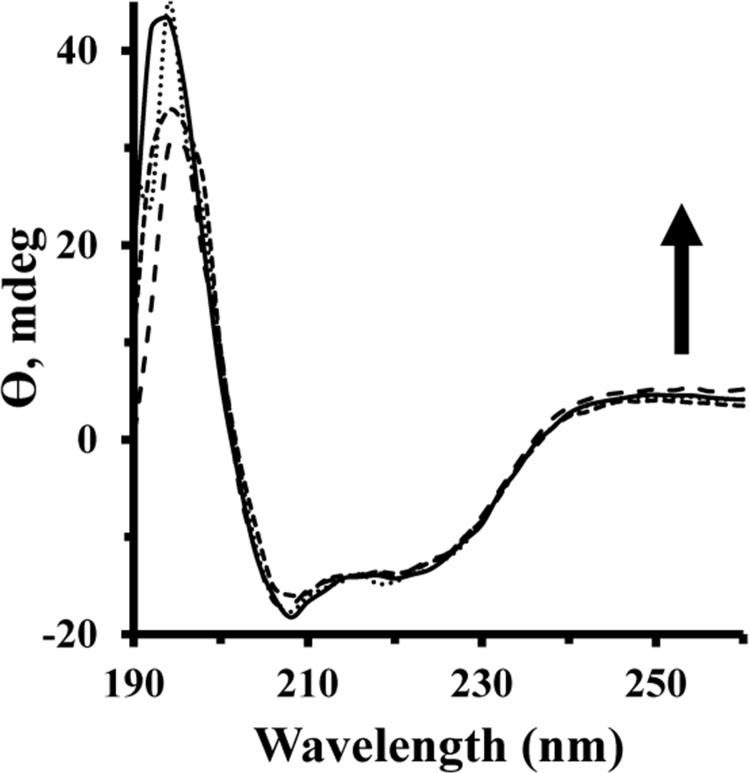
Far UV-CD spectra of HEWL upon addition of Fe NP. The secondary structural changes of HEWL with a concentration of 15 μM (pH 7.8, 50 mM phosphate buffer) were monitored in the presence of varying concentrations (5–150 μM) of Fe NP.

Circular dichroism (CD) spectroscopy has been employed as a pivotal technique in structural biology investigation to explore proteins structural changes upon interaction with ligands [35, 36]. This method has been shown to provide sufficient and reliable evidence of protein conformational changes. Typically, the CD spectra of proteins are monitored in the far-UV wavelengths (190–260 nm) and the near-UV wavelengths (260–320 nm) to consider secondary and tertiary structural changes of proteins, respectively. The CD spectra of HEWL showed two negative minima at 208 nm and 222 nm, corresponding to dominant helical structure of protein [37, 38]. Fig 8 demonstrates that the minimum intensity of HEWL has not been altered in the presence of varying concentrations of Fe NP at room temperature. These outcomes indicate that the binding of Fe NP has not influenced the secondary structure of HEWL.

**Fig 9:**
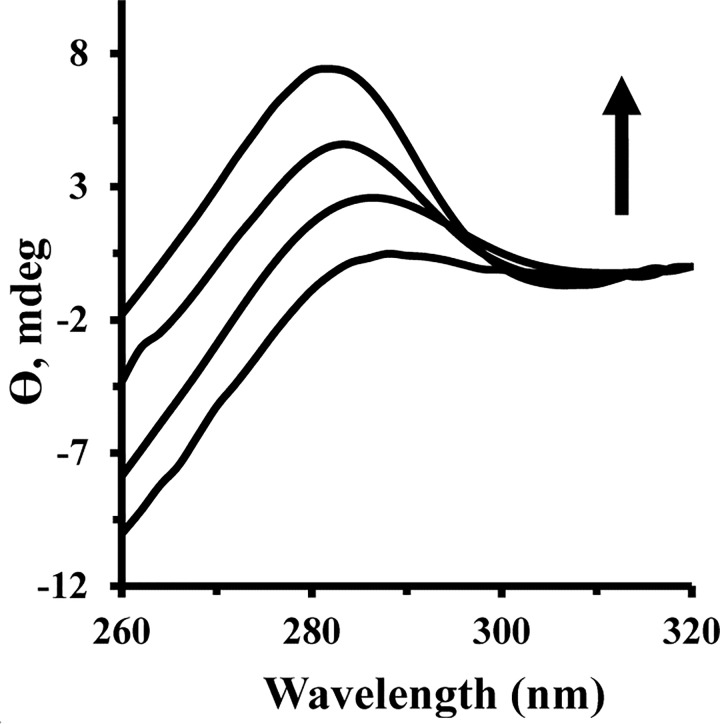
Near UV-CD spectra of HEWL upon addition of Fe NP. Near CD spectra of HEWL (75μM) were monitored in the presence of varying concentrations of Fe NP (25–75 μM).

Fig 9 shows the changes in HEWL tertiary structure upon interaction with Fe NP. Near-UV CD provides decisive detail regarding the disposition of aromatic residues in the protein and is dramatically sensitive to interactions between choromophores and ligand. Fig 9 shows a gradual increase in peak intensity of HEWL about 280–290 nm, which is attributed to the tertiary structural changes of HEWL after addition of Fe NP. Also a remarkable blue shift was observed for maximum absorption wavelength of HEWL after addition of Fe NP, which brings complementary outcomes to the structural unfolding of protein and corresponding exposure of aromatic residues to the solvent. This result is in good agreement with synchronous fluorescence spectroscopy and ANS-fluorescent experiments. Indeed, the established polar-polar interactions between polar residues of protein surface and water molecules on NP surface are not strong enough to perturb the secondary structure of protein, whereas, these interactions can replace some interamolecular polar bonds with intermolecular ones and extend the protein structure to a more unfold one.

### Molecular docking study

Molecular docking studies play an important role in molecular biology study and drug discovery. Computational docking studies were used after experimental measurements to further analyze Fe NP–HEWL interactions and determine the contribution of site specific binding amino acids. Therefore, the possible conformations of the Fe NP–HEWL complex were calculated using HEX 6.3 software [13]. The X-ray crystallographic 3D structure of HEWL (PDB ID: 6lys) was downloaded from the online protein data bank (http://www.pdb.org). HEWL consists of 129 amino acids containing five helices and five beta-strands, which are arranged in two antiparallel sheets. The molecular docking was performed in the presence of water-coated nanoparticle. The resulting binding energies were found to be -230.92 kJ/mol. This value shows the high binding affinity for decorated nanoparticle to the protein.

**Fig 10:**
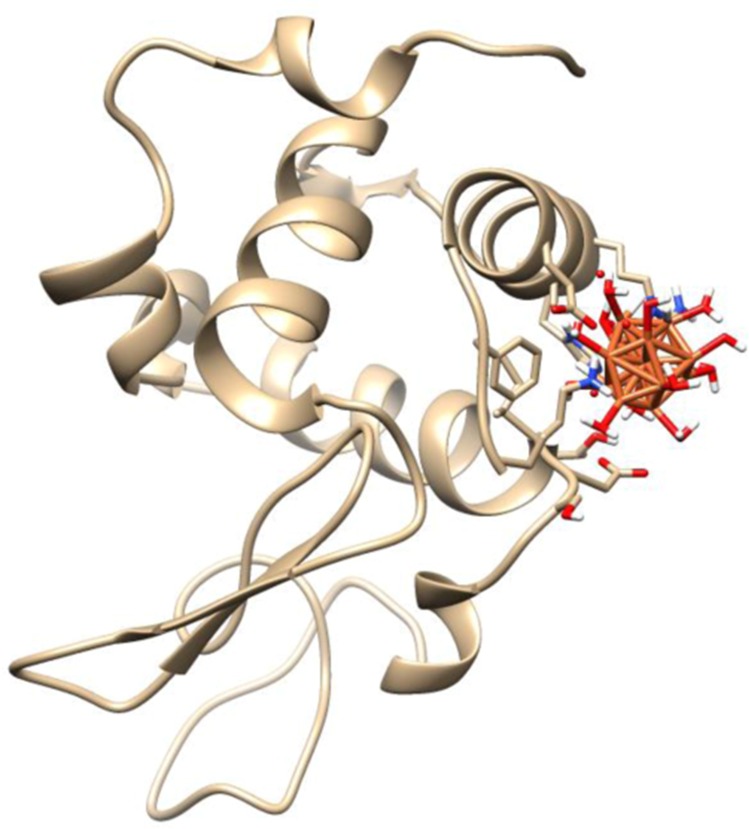
Molecular docking study. Molecular docking of the HEWL with water-coated Fe NP.

**Fig 11:**
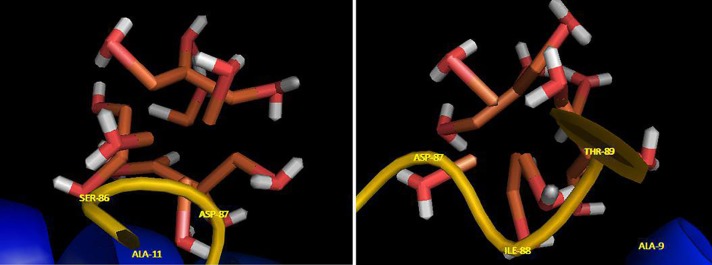
Fe NP binding site. The ligand and its surrounding site residues at two rotational view.

Visualization of the docked pose has been done by using CHIMERA (www.cgl.ucsf.edu/chimera) and PyMOL (http://pymol.sourceforge.net/) tools. The docked complex is shown in Fig 10. The ligand with surrounding active site residues within 3.5 Å and the spatial orientation in binding pocket is given in Fig 11. The closest interacting residues which can contribute in hydrogen bonding are SER-86, ASP-87, ILE-88, and THR-89. The hydroxyl and carboxyl groups provide a prepared medium to make hydrogen bonds with water molecules on iron surface. The average binding energy per amino acid is approximately -58 kJ/mol that reveals a moderate hydrogen bond for each amino acid.

## Conclusions

The interaction of positively charged HEWL and Fe NP has been occurred mainly by hydrogen bonds due to presence of polar residues on the surface of protein and a water shell around Fe NP surfaces. Our results demonstrated that there is approximately half binding site on HEWL for Fe NP. Based on the thermodynamic parameters, the binding mode of Fe NP to HEWL is an enthalpy-driven and exothermic process which hydrogen bonding and van der Waals interaction play considerable roles in the formation of protein-nanoparticle system. From fluorescence and circular dichroism spectroscopy outcomes, it can be concluded that after addition of Fe NP to HEWL, conformational changes at the level of tertiary structure can occur and secondary structure of protein is preserved. Also it was shown the adsorbed proteins change the zeta potentials and the isoelectric points (IEP) of the particles.

Interaction of Fe NP with HEWL induces tertiary structural changes of protein while the secondary structure of protein remains almost constant. However the degree of introduced conformational change is not much enough to surpass the potential energy barriers of hydrophobic moiety of protein. Hence nanoparticles may not easily reach the hydrophobic interior of proteins and choromophore residues existing almost close to the protein surface are prone to be affected by interaction with small molecules. HEWL contains six Trp (28, 62, 63, 108, 111, and 123) and three Tyr (20, 23, and 53) residues in its sequence. Trp and Tyr residues are not equally accessible to the protein surface and detection of intrinsic fluorescence intensity of protein provides potent details in uncovering the disposition of Trp and Tyr residues in protein conformational changes. Conformational changes of HEWL may be induced by Fe NP binding. Charged Fe NP normally attach to the complementary charged residues on the surface of proteins. In this case, positively charged HEWL and Fe NP establish hydrogen bonds instead of electrostatic interactions. It may be explained that these two particles present positive charges, and they are not able to establish a potent electrostatic interaction. Therefore it may be suggested that the zeta potential values of HEWL and Fe NP are not high enough to create the electrostatic interaction in the presence of water. In other words, when the zeta potential is low, hydrogen bonds may exceed the electrostatic interactions and the protein/nanoparticle complex may be formed.

Here we described a nanoparticle-protein interaction system that induces protein structural changes in response to changes in the surface environment rather than charge of protein and nanoparticle surface. The binding of water molecules to the surface of particles at ambient temperature results in a considerable surface modification, significantly changing the orientation of water molecules of the surface relative to bulk molecules and finally to form a surface with abundant hydroxyl moieties. These ideas imply a mechanism for better understanding of nanoparticle-induced protein conformational change and the potential application of the nanoparticle structure. Furthermore, the results suggest that the reactivity of nanoparticles surfaces, in biological application will be determined by both particle charge and the characteristics of the surrounding molecules. The mechanism of interaction of HEWL with Fe NP was shown to result in a negative entropy changes. In this case, the increase in entropy of released water molecules on the nanoparticle and protein surfaces is smaller than the decrease in the entropy of attached proteins. In other world, a wide number of water molecules are involved in the hydrogen interactions between surface of protein and nanoparticle. Taken together, it can be concluded that Fe NP shows a major effects on the tertiary structure of HEWL. Hence, for medical use of Fe NP, it may be required more toxicity assays before expanding their application in cellular and subcelluar systems.
